# Micro-structural bone changes in early rheumatoid arthritis persist over 1-year despite use of disease modifying anti-rheumatic drug therapy

**DOI:** 10.1186/s12891-017-1888-3

**Published:** 2017-12-11

**Authors:** Lynne M. Feehan, Linda L. Li, Heather A. McKay

**Affiliations:** 1 Department of Physical Therapy, University of British Columbia, Vancouver, BC Canada; 2 Arthritis Research Canada, 5591 No. 3 Road, Richmond, BC V6X 2C7 Canada; 3 Department of Orthopedics and Family Practice, Centre for Hip Health and Mobility, University of British Columbia, Vancouver, BC Canada

**Keywords:** High resolution – Peripheral quantitative computed tomography (HR-pQCT), Early rheumatoid arthritis, Disease modifying Antirheumatic drugs, Bone health, Osteoporosis, Fracture risk

## Abstract

**Background:**

We used High Resolution – peripheral Quantitative CT (HR-pQCT) imaging to examine peri-articular bone quality in early rheumatoid arthritis (RA) and explore whether bone quality improved over 12-months in individuals receiving care consistent with practice guidelines.

**Methods:**

A 1-year longitudinal cohort study (Baseline and 12-months) evaluating individuals with early RA compared to age/sex-matched peers. Personal demographic and health and lifestyle information were collected for all. Whereas, active joint count (AJC_28_), functional limitation, and RA medications were also collected for RA participants. HR-pQCT imaging analyses quantified bone density and microstructure in the Metacarpal Head (MH) and Ultra-Ultra-Distal (UUD) radius at baseline and 12-months. Analyses included a General Linear Modelling repeated measures analyses examined main effects for disease, time, and interaction on bone quality.

**Results:**

Participants (*n* = 60, 30 RA/30 NRA); 80% female, mean age 53 (varying from 21 to 74 years). At baseline, RA participants were on average 7.7 months since diagnosis, presenting with few active joints (AJC_28_: 30% none, remaining 70% Median 4 active joints) and minimal self-reported functional limitation (mHAQ-DI_0–3_: 0.56). At baseline, 29 of 30 RA participants had received one or more non-biologic disease-modifying anti-rheumatic drugs (DMARD);13 in combination with glucocorticoid and 1 in combination with a biologic medication. One participant only received glucocorticoid medication. Four RA participants withdrew leaving 26 pairs (*n* = 52) at 12-months; 23 pairs (*n* = 46) with UUD and 22 pairs (*n* = 44) with MH baseline and 12-month images to compare. Notable RA/NRA differences (*p* < 0.05) in bone quality at all three sites included lower trabecular bone density and volume, more rod-like trabeculae, and larger and more variable spaces between trabeculae; fewer trabeculae at the UUD and MH2 sites; and lower cortical bone density and volume in the MH sites. Rate of change over 12-months did not differ between RA/NRA participants which meant there was also no improvement over the year in RA bone quality.

**Conclusions:**

Early changes in peri-articular bone density and microstructure seen in RA are consistent with changes more commonly seen in aging bone and are slow or resistant to recover despite well controlled inflammatory joint symptoms with early DMARD therapy.

**Electronic supplementary material:**

The online version of this article (10.1186/s12891-017-1888-3) contains supplementary material, which is available to authorized users.

## Background

Rheumatoid arthritis (RA) affects 1% of adults, most commonly in women (3:1) aged 40 to 70 years [1]. Despite marked improvement in the clinical management of progressive joint disease, individuals with RA continue to live with underlying bone changes and are twice as likely to sustain any fracture compared to people without RA [2–6]. The underlying mechanism(s) for changes in bone health in RA are likely multi-factorial. These may include: 1) response to local inflammatory cytokines, hypervascularity or bone edema in the periarticular bone adjacent to inflamed joints [7], 2) systemic inflammatory mediated catabolic imbalance in normal bone homeostatic mechanisms [8], 3) bone adaptive resorption in response to physical inactivity [9, 10], and 4) effect of RA medications [11–13].

Clinically, radiographic (x-ray) progression of periarticular osteopenia, joint space narrowing and focal bony erosions, primarily in the hand and wrist, are considered the hallmark of poor disease control [14]. Magnetic resonance imaging, computed tomography, dual x-ray absorptiometry and digital x-ray radiogrammetry can also identify *macro* structural bone changes in RA [15–17]. However, these clinical imaging technologies are unable to evaluate *micro* structural adaptations that accompany bone and joint diseases. High Resolution – peripheral Quantitative CT (HR-pQCT) is a reliable and precise imaging system that detects and quantifies micro-structural bone alterations, and can do so before macro-structural changes appear [18]. Specifically, standardized protocols for HR-pQCT image acquisition and evaluation methods in RA permit characterization of periarticular trabecular and cortical bone volumetric density and microstructure in the periarticular regions of the metacarpal head (MH2,3) and distal radius (UUD - ultra-ultra-distal) [19, 20].

To date, only a small number of cross-sectional HR-pQCT studies evaluated periarticular bone quality in those with RA [21–27]. Notably, there was consistent evidence of changes in periarticular bone density and microstructure in the Metacarpal Head (MH) or Distal Radius in individuals living with RA for 8 or more years, [21–26], as well as, emerging evidence of early bone changes potentially occurring before the onset of inflammatory joint symptoms [27]. However, given the cross-sectional design and heterogeneity of RA participants in these previous studies it was not possible to define *when* changes in bone micro-structure occurred in relation to RA onset or response to RA treatments.

We aimed to fill this gap by examining periarticular bone density and microstructure adaptations in patients with early RA who receive care consistent with current clinical guidelines [28–30]. Specifically, the purpose of this study was to use HR-pQCT to examine: 1) differences in trabecular and cortical bone density and microstructure in MH and DR periarticular bone in individuals with early RA (< 1 year) and started on Disease Modifying Antirheumatic Drug (DMARD) therapy at the time of diagnosis, and 2) whether bone density and microstructure changes improve, persist or deteriorate over 12-months. We hypothesized that we would identify early microstructural damage within a year of RA diagnosis and that early micro-structural bone damage would persist (not improve or worsen) despite adequate disease control with first line DMARD [+/- Glucocorticoid (GC)] therapies over the subsequent 12-months.

## Methods

We conducted a one-year, prospective observational cohort study in individuals living independently in the community in a large urban metropolitan region (Greater Vancouver Regional District, British Columbia, Canada). All participants were 19 years or older and provided informed consent. RA participants had to be treated by a rheumatologist and have a rheumatologist-confirmed diagnosis of new onset RA (< 1 year) based on the American College of Rheumatology / European League Against Rheumatism (ACR/EULAR) 2010 criteria [31]. Individuals were excluded if they had any health condition that prevented participation, had metal or surgical implants in their dominant arm, were pregnant, had sustained a fracture in their dominant arm in the previous 12 months, or were unable to provide consent. Non-RA (NRA) participants were also excluded if they had been told by a physician they had any inflammatory joint disease or rheumatologic condition. Patients diagnosed with new onset RA were identified from nine rheumatology clinics and the research team confirmed eligibility. For comparison, sex and age-matched NRA participants were recruited through word of mouth, flyer postings in health care settings, email requests and research website postings. NRA participants were screened for eligibility and were matched with an RA study partner by sex and age within 2 years.

### Evaluations

**Fig. 1:**
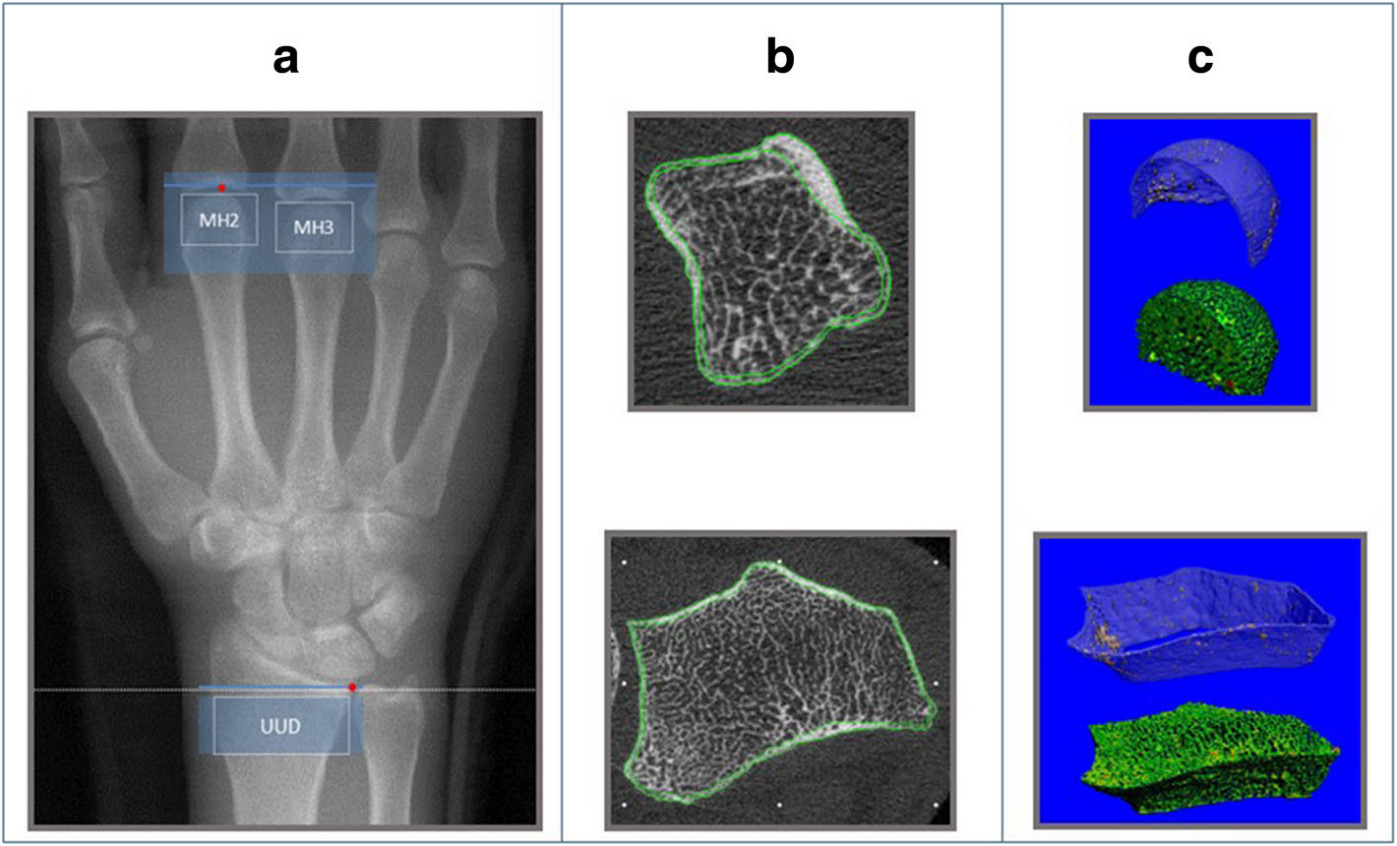
**a** 150 mm Scout View Image. Dots = Reference points for the distal radial (medial distal cortex radius) and metacarpal head (distal tip of most proximal MH) scans. Larger shaded boxes = Distal Radius (110 slices) and Metacarpal (220 slices) scan lengths. Smaller boxes with UUD, MH2 and MH3 text = Three Regions of Interests (ROIs) evaluated (UUD = Ultra-ultra distal, MH = Metacarpal head 2 or 3). **b** Single HR-pQCT image slices of a cross-sectional image of MH (Top) and Distal Radius (Bottom) scans. Lines show cortical bone periosteal and endosteal semi-automatic segmentation. **c** 3-Dimensional reconstructed images of MH (top) and UUD (bottom) scans of a non-RA participant, with the cortical bone and trabecular bone regions separately reconstructed

Participants attended baseline and 12-month in-person evaluations. ***Physical Evaluations:*** Measures of height (cm) and weight (kg) [Body Mass Index (BMI): Kg / m2] were collected for all participants and a 28-joint active (Tender and Swollen) joint count for RA participants [32]. ***Self-Reported Measures***: Participants completed a General Health and Lifestyle questionnaire. RA participants also completed a Stanford Health Assessment Questionnaire - Modified (mHAQ) [33]. ***HR-pQCT Imaging:*** The imaging protocol has been described in detail elsewhere [19]. Briefly, we acquired HR-pQCT images with a Scanco XtremeCT imaging system [Scanco Medical AG, Switzerland] using standard manufacturer recommended parameters [19]. For the radius, the reference line was the medial/distal radius (Fig. 1a – Scout View). The scan started 3 mm proximal to this reference line and extended 9.02 mm (110 slices) proximally (Fig. 1a – Scout View). For the metacarpal head the reference line was the tip of the most distal second or third metacarpal head. The scan started 4 mm distal to this reference line and extended 18.04 mm (220 slices) proximally [19, 20]. (Fig. 1a -Scout View). Each 110-slice scan takes 2.8 min with an effective dosage of less than 2 μSv [19].

### Medical record/medication data

At baseline an internal medicine resident extracted information from rheumatologists’ electronic medical records including; RA diagnosis date, timing and type of prescribed RA medication(s), and RA blood markers [anti-citrullinated peptide autoantibodies (ACPA) and/or Rheumatoid Factor (RF) Positive] at time of diagnosis. At 12-months, RA participants completed a log of RA medications prescribed and taken in the previous 6 months. The cumulative dosage of any DMARD or GC medications were not collected at either time point.

### HR-pQCT image analyses

One of three trained operators [intra-rater reliability, 10 scans measured twice, Pearson’s *r* > 0.9] analyzed all images using manufacturer’s evaluation software (SCANCO, V 6.0) [19]. Prior to analysis, each image was graded for motion artifact using a 5-point grading scale and only images rated 3 or higher were used [19]. Regions of Interest included the ultra-ultra-distal radius (UUD: 110 slices, starting 3 mm proximal to radius reference line and running proximally), metacarpal head two (MH2: 110 slices starting at the distal tip of MH2 running proximally), and metacarpal head three (MH3: 110 slices starting at the distal tip of MH3 running proximally) (Fig. 1a – Scout View) [19]. We ran standard manufacturer and direct transformation image analyses scripts to segment the cortical and trabecular bone regions and measure bone density and microstructure (Fig. 1b, c – Cortical/Trabecular Compartment Segmentation) [19].

Density measures included apparent bone mineral density (BMD - mgHA/cm^3^) for cortical and trabecular bone regions, and cortical bone material bone density (TMD - mgHA/cm^3^). Measures of cortical microstructure included: thickness (CtTh - mm), thickness variability CtThSd - mm), volume fraction (BV/TV_cort_ - %) and porosity (CtPo - %). Measures of trabecular microstructure included: volume fraction (BV/TV_trab_ - %), number (TbN – 1/mm), thickness (TbTh - mm), separation (TbSp - mm), separation variability (TbSpSd - mm), connective density (TbCD - mm^4^) and structural model index (SMI _0–3_; lower values indicate more plate-like verses more rod-like structure) [19].

### Statistical analyses

We examined differences in baseline anthropometrics (Age, Sex, BMI) and Fracture Risk between RA and NRA participants using Paired Student T-tests (two tailed, *p* < 0.05). For the longitudinal analyses of micro-structural bone quality, we conducted a General Linear Modelling 2 × 2 repeated measures analyses for the HR-pQCT image analyses. We compared disease status (RA vs NRA) over Time [Baseline vs 12 Month] using a two-tailed analysis with a Sidak multiple comparison adjustment. We set alpha at *p* = 0.05. We examined main effects for disease (RA vs NRA, independent of time), time (change over 1-year, independent of disease status) and interaction [disease (RA vs NRA) x time (baseline vs 12- months)]. We did not correct for separate statistical analyses for multiple HR-pQCT imaging outcomes. All statistical analyses were completed using SPSS software v. 23 (IBM Corp, Armonk, NY).

## Results

### Recruitment/retention

Forty-nine individuals diagnosed with RA in the previous year by one of nine rheumatologists were screened for eligibility, with 19 excluded (39% excluded). Reasons for exclusion were RA diagnosis at the time of screening greater than 1 year (*n* = 10), not able to commit to study (*n* = 6), or language barrier (*n* = 3). Of 43 NRA individuals screened eight were excluded (19% excluded) because they were unable to commit to the study (*n* = 5) or they reported a co-morbid inflammatory health condition (n = 3). Of the 35 eligible NRA participants five were ultimately excluded as they were not matched with an RA study partner. The 60 participants were serially recruited to the study and evaluated at baseline over an 11-month time period. Fifty-six participants (26 RA/30 NRA) completed the 12-month evaluation (93% completion); 54 were evaluated at 52-weeks (+/− 4 weeks) and 2 were evaluated at 60 weeks. Four RA participants withdrew [1 death, 2 serious illness (cancer, cardiac disease), 1 no longer interested] and their 4 sex/age-matched NRA partners were excluded from the final longitudinal analyses. Five images were excluded from analyses due to motion artifact at baseline (4.2%; 2-MH, 3-UUD) and at 12-months (9.6%; 4-MH, 1-UUD). Of the 26 matched pairs of participants at 12 months (*n* = 52); 23 pairs (88.5%; *n* = 46) had baseline and 12-month UUD images and 22 pairs (84.6%; *n* = 44) had baseline and 12-month MH images for comparison.

### Participant demographics

See Table 1 for further details of participant demographics at baseline. In summary, of the 60 participants, 48 were females with a mean age of 53 years varying from 21 to 74 years. The RA and NRA pairs were well matched by age and sex, with exact matching for sex and no statistically significant difference in age, with the RA participants on average 53 years old, compared to the NRA participants 52 years old. On average, female RA and both RA and NRA males were overweight (BMI: 25–29.9 kg/m^2^), whereas, NRA females were of high normal body weight (BMI 24.8 kg/m^2^). [34] The mean BMI was significantly higher in the RA group for women, but not for men. The mean Fracture Risk Assessment Tool (FRAX®_Canada_) 10-year major fracture and hip fractures risk scores were both significantly higher among participants with RA [35]. Some other differences at baseline included, 6 RA participants smoked compared to 1 NRA participant, 7 RA participants compared to 1 NRA participant had been told they may have poor bone health (i.e. osteopenia or osteoporosis) and 3 individuals with RA reported taking a bone antiresorptive or anabolic medication in the last 5 years [36]. As well, 19 RA participants reported taking calcium or vitamin D nutritional supplements compared with 13 of NRA participants.

**Table 1:** Baseline Demographics: Rheumatoid Arthritis vs Non-Rheumatoid Arthritis Participants (*n* = 60)

Domain	Parameter	RA (*n* = 30)	NON-RA (n = 30)
Age	Age in Years [mean (SD), min-max]	53.3 (13.7), 21–74	51.6 (13.6), 23–70
Sex	Sex [# (%) - Male, Female]	6 (20%), 24 (80%)	6 (20%), 24 (80%)
Body Mass Index (BMI)	BMI Female [mean (SD), min-max]	**28.3 (7.9), 16.8–49.3**	**24.3 (4.8), 18.9–37.7**
	BMI Male [mean (SD), min-max]	27.5 (4.1), 20.1–32.5	26.8 (2.5), 24.1–29.9
Fracture Risk – FRAX ® [35]	10 year - Major Fracture Probability (%) - FRAX (Canada), no aBMD [mean (SD), min-max]	**11.2 (9.9), 1–43**	**5.9 (4.9), 1–18**
	10 year - Hip Fracture Probability (%) - FRAX (Canada), no aBMD [mean (SD), min-max]	**3.2 (4.2), 0–17**	**1.0 (1.3), 0.1–5.5**
Bone Health - Risk Factors (Self-Report)	Current Smoker [# (%)]	6 (20%)	1 (3%)
	*Current Alcohol (0 to 4, higher score more alcohol consumption) [median, mode (%)]	1, 1 (30%)	2, 1 (37%)
	Told in the last five years by any physician that they (may) have osteoporosis [# (%)]	7 (23%)	1 (3%)
Bone Health - Medications/Nutritional Supplements (Self-Report)	Bone Antiresorptive or Anabolic Medication - Last 5 years [#, (%)]	3 (10%)	0 (0%)
	Current Calcium, Vitamin D Supplement Intake [#, (%)]	19 (63%)	13 (43%)

*Current Alcohol Use (alcohol drinks / week): ‘0’ none, ‘1’ <1, ‘2’ 1 to 3, ‘3’ 4 to 7, ‘4’ >7

aBMD = Apparent Bone Mineral Density measured by DXA

Bold indicates a statistically significant difference between RA and NRA participants (Two tailed, Paired Student T-test)

### RA participant disease characteristics

See Table 2 for further details of RA participant characteristics. In summary, at baseline the 30 RA participants were on average 7.7 months (varying from 1 to 15 months) since diagnosis. Notably, one RA participant was scanned at 15-months post diagnosis which was a protocol violation. This occurred as the participant did attend the baseline evaluation at 12-months post diagnosis, however, they could not complete the evaluation due to physical illness. Unfortunately, the re-evaluation was subsequently delayed due to a 3-month planned vacation. Twenty-two of the RA participants were ACPA and/or RF positive at the time of diagnosis. At baseline, 29 of 30 RA participants received one or more non-biologic DMARD, including Methotrexate, Hydroxychloroquine, or Sulfasalazine. Of these, 14 received single, 7 received double and 8 received triple DMARD therapy. Thirteen individuals received only DMARD therapy, 15 received DMARDs in combination with oral glucocorticoid (at least one episode of Prednisone, > 5 mg/day, > 3 weeks), 1 received DMARD in combination with an anti-TNF (Tumor Necrosis Factor) biologic medication (Adalimumab) started 8-months post diagnosis and 1-week prior to baseline imaging and 1 person having only received glucocorticoid medication. DMARD therapies were started on average 0.1 month after diagnosis, whereas, oral glucocorticoid (GC) medication was initiated on average 1.8 months prior to final RA diagnosis. The negative value for onset of GC medication can be explained in part by a typical 4 to 6-week time interval between the initial visit to the rheumatologist and final diagnosis of RA, when GC medications are often started prior to definitive RA diagnosis. Additionally, one participant had received a GC medication prescription (> 5 mg/day for 3 weeks) from their primary care physician 11-months prior to the referral to a rheumatologist. At 12-months, 24 of 26 RA participants were still receiving DMARD therapy. However, only 2 had taken any oral glucocorticoid medications in combination with DMARDs in the previous 6 months and 19 had tapered down to single DMARDs therapy. Whereas, one person was taking only an anti-TNF biologic medication (Adalimumab), while another had opted out of taking all prescribed RA medications as a personal choice.

**Table 2:** Rheumatoid Arthritis (RA) Participant Clinical Characteristics

Domain	Parameter	RA Baseline (*n* = 30)	RA 12-Months (*n* = 26)
RA Diseases Duration	Months Since Diagnosis by a Rheumatologist [mean (SD), min-max]	7.7 (4.9), 1–15*	Baseline Only
Rheumatoid Arthritis Blood Markers	Anti-cyclic citrullinated protein antibodies (anti-CCP) and/or Rheumatoid Factor (RF) Positive [# (%)]	22 (73%)	Baseline Only
RA Medication Combinations	**8 DMARD Only [# (%)]	13 (43%)	22 (85%)
	DMARD + Glucocorticoid [# (%)]	15 (50%)	2 (7%)
	DMARD + Biologic [# (%)]	1 (3%)	0 (0%)
	DMARD + Glucocorticoid + Biologic [# (%)]	0 (0%)	0 (0%)
	Single DMARD [# (%)]	14 (47%)	19 (73%)
	Double DMARD [# (%)]	7 (23%)	4 (15%)
	Triple DMARD [# (%)]	8 (27%)	1 (4%)
	*** Glucocorticoids Only [# (%)]	1 (3%)	0 (0%)
	**** Biologic Only [# (%)]	0 (0%)	1 (4%)
	***** No RA medications [# (%)]	0 (0%)	1 (4%)
RA Medication Timing	Months to any DMARD once Diagnosed (*n* = 29) [mean (SD), min-max]	0.1 (1), −4 to 3	Baseline Only
	Months to Glucocorticoids Once Diagnosed (*n* = 16) [mean (SD), min-max]	−1.8 (3), −11 to 1	Baseline Only
	Months to Biologic Once Diagnosed (n = 1)	8	Baseline Only
Physical Evaluation – 28-Joint Active (Tender AND Swollen) Joint Count	Number participants with NO Tender AND Swollen Joints [*n* (%)]	9 (30.0)	15 (57.7)
	Number participants with at least one Tender AND Swollen Joint [*n* (%)]	21 (70.0)	11 (42.3)
	Number of Tender AND Swollen Joints [mean (SD), min-max]	4.2 (2.3), 1–9	3.6 (3.0), 1–9
Stanford Health Assessment Questionnaire - Modified (MHAQ) [33]	Disability Index - 0 to 3 [mean (SD), min-max]	0.59 (0.60), 0 to 2.13	0.48 (0.66), 0 to 2.00
	Pain Visual Analog Scale (VAS) - 0 to 100 [mean (SD), min-max]	21.2 (16.4), 0 to 65	20.4 (23.3), 0 to 89
	Global Functioning VAS - 0 to 100 [mean (SD), min-max]	23.7 (18.8), 1 to 68	18.2 (21.7), 1 to 88

*One RA participant received baseline HR-pQCT scan at 15-months post-diagnosis. The participant attended the baseline evaluation at 12-months post diagnosis, but was sick and could not be re-scheduled due to a planned 3-month vacation

**Non-Biologic Disease-Modifying Anti-Rheumatic Drug (DMARD): Methotrexate, Hydroxychloroquine, Sulfasalazine

***Glucocorticoid (GC): > = 5 mg / day for ≥3 weeks

****Biologic anti-TNF: Adalimumab

*****One person moved to an alternative medicine practitioner and stopped all RA prescribed meds

NOTE: Cumulative dosage for any RA medications and medication adherence were was not tracked

### RA participant clinical characteristics

See Table 2 for further details of RA participant clinical characteristics. This study used a 28-joint active count as a measure for evidence of active joint inflammation (i.e. no or some active joints) at the time of imaging, with active inflammation of any joint defined as both swollen and tender. At baseline, 9 of 30 RA participants presented with no active joints. Of the remaining 21 RA participants with at least one active joint, the median active joint count was 4. At 12-months, 15 of 26 RA participants presented with no active joints. Of the remaining 11 participants with at least one active joint, the median active joint count was 1. At baseline RA participants reported low levels of functional disability [DI_1–3_: Mean 0.59 (+/−0.60)], pain (VAS_0–100_: Mean 21.2 (+/− 16.4) and impact on well-being [VAS_0–100_: Mean 23.7 (+/− 18.8)] [33]. Notably, RA participants also reported minimal improvement in their self-reported functional disability, pain or impact on well-being over 1-year [37].

### HR-pQCT imaging – Disease (RA vs NRA) effects

**Fig. 2:**
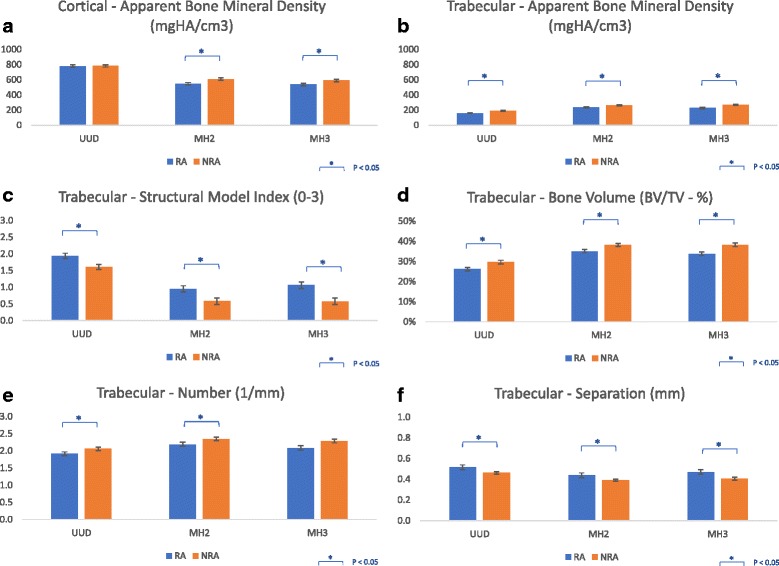
Common Changes in Density and Micro-structure across Regions of Interest: Bar graphs [mean (SEM)] showing differences between rheumatoid arthritis (RA) and Non-RA (NRA) participants for selected density and microstructural variables with consistent differences across two or three of the regions of interest examined in this study. This figure also illustrates regional differences density and microstructural in the two MH compared to the UUD ROIs

**Fig. 3:**
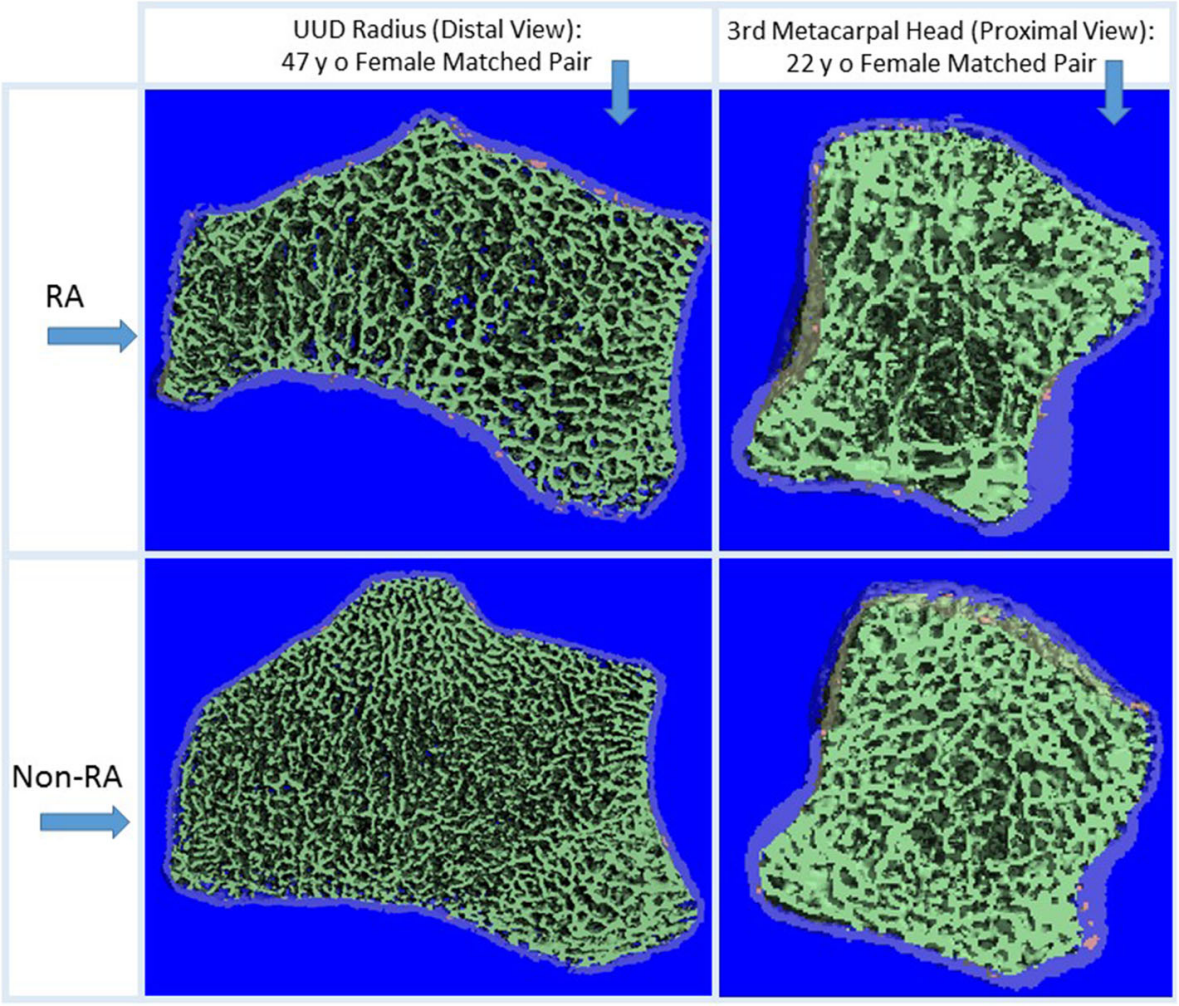
Examples of cross-sectional reconstructions from UUD radius scans (distal view - left column) from two 47-year-old women who were RA and NRA matched study partners and MH3 scans (proximal view – right column) from two 22-year-old women who were RA and NRA matched study partners. The reconstructed images in the top row are from the RA participants and the reconstructed images in the bottom row are from the NRA participants. Images illustrate marked visual differences in trabecular bone microstructure between the RA and NRA participants. The 3-D reconstructions show the peripheral cortical region in lighter shading compared to the central trabecular region (darker shading)

See Table 3 for details of the values and results of the longitudinal statistical analyses for the HR-pQCT disease main effect, representing the overall differences between RA and NRA participants independent of time at all three sites. In summary, *differences in density included*: RA participants had significantly lower trabecular bone density at all three sites (varying from 15.4 to 10.9% lower), significantly lower cortical bone apparent density at both MH sites (MH2 8.7% and MH3 9.9% lower) and significantly lower cortical bone material density at the MH2 site (MH2 3.9% lower). *Differences in cortical bone micro-structure included:* At the UUD site RA participants demonstrated significantly greater variability in cortical thickness (9.3% greater variability in thickness) and significantly less cortical bone volume at both MH sites (MH3: 3.8% MH2: 4.5% lower). *Trabecular bone micro-structure differences included:* At all three sites RA participants had significantly larger and more variable sized spaces between trabeculae (varying from 11.9% to 16.0% larger spaces and 22.9% to 35.2% more variable sized spaces). At all three sites RA participants trabecular matrix was also significantly more rod- verses plate-like shaped trabecular matrix (SMI varied from 20.8% to 84.1% greater) with significantly lower trabecular bone volume (varying from 12 to 8% lower). At the UUD and MH2 sites, RA participants also had significantly fewer trabeculae (UUD 7.2% lower; MH2 9.0% lower). Whereas, at the UUD site RA participants also had trabecular that were less connected (13.0% less trabecular connectivity). Figure 2 shows plots (mean +/− SEM) for selected density and microstructural variables with consistent differences between RA and NRA participants across the three ROIs examined in this study, as well as, notable differences in the density and micro-structure values of the two MH head ROIs and the UUD ROI. To illustrate further the differences between RA and NRA participants, Fig. 3 shows the typical visual differences in trabecular bone micro-structure seen in 3-Dimensional reconstructed images of the UUD and MH sites in age- and sex-matched RA and NRA study partners.

**Table 3:** HR-pQCT Disease (Rheumatoid Arthritis vs No Rheumatoid Arthritis) Main Effects Summary

Region of Interest (ROI)	Parameter	Outcome	Variable	RA Participants		NRA Participants		*P* Value	Group Diff Value (%)
				Mean	SEM	Mean	SEM	(2-tailed)	(RA-NRA)
UUD (n = 46, 23 pairs)	Volumetric Bone Density	Cortical Material Bone Mineral Density (mgHA/cm^3^)	TMD_cort_	946.54	8.61	945.36	7.67	0.903	1.19 (0.1%)
		Cortical Apparent Bone Mineral Density (mgHA/cm^3^)	BMD_cort_	779.71	15.64	782.14	14.98	0.889	−2.43 (−0.3%)
		Trabecular Apparent Bone Mineral Density (mgHA/cm^3^)	BMD_trab_	159.84	6.99	188.15	7.19	**0.001**	**−28.31** **(−15.0%)**
	Cortical Microstructure	Cortical Thickness (mm) - Direct	CtTh	0.73	0.03	0.69	0.03	0.241	0.05 (6.7%)
		Cortical Thickness _ SD (mm)	CtThSd	0.30	0.01	0.27	0.01	**0.047**	**0.03 (9.3%)**
		Cortical Bone Volume Fraction (%)	BV/TV_cort_	91.55%	0.76%	91.56%	0.98%	0.993	−0.01 (0.0%)
		Cortical Porosity (%)	CtPo	1.76%	0.22%	1.71%	0.24%	0.830	0.04 (2.6%)
	Trabecular Microstructure	Trabecular Bone Volume Fraction (%) -Direct	BV/TV_trab_	26.08%	0.92%	29.66%	0.88%	**0.001**	**−3.57** **(−12.1%)**
		Trabecular Connective Density (mm^4^)	TbCD	4.14	0.22	4.76	0.20	**0.005**	**−0.62** **(−13.0%)**
		Trabecular Structural Model Index (0–3)	SMI	1.94	0.08	1.61	0.08	**0.002**	**0.33 (20.8%)**
		Trabecular Number (1/mm)	TbN	1.91	0.06	2.06	0.05	**0.016**	**−0.15** **(−7.2%)**
		Trabecular Thickness (mm) - Direct	TbNSd	0.195	0.002	0.199	0.002	*0.130*	*−0.005* *(−2.3%)*
		Trabecular Thickness _SD (mm)	TbTh	0.07	0.00	0.07	0.00	0.419	0.001 (1.9%)
		Trabecular Separation (mm)	TbSp	0.52	0.02	0.46	0.01	**0.006**	**0.05 (11.9%)**
		Trabecular Separation_SD (mm)	TbSpSd	0.22	0.02	0.18	0.01	**0.023**	**0.04 (22.9%)**
MH3 (n = 44, 22 pairs)	Volumetric Bone Density	Cortical Material Bone Mineral Density (mgHA/cm^3^)	TMD_cort_	815.17	10.67	840.60	9.70	*0.055*	*−25.43* *(−3.0%)*
		Cortical Apparent Bone Mineral Density (mgHA/cm^3^)	BMD_cort_	537.77	15.52	589.12	16.17	**0.012**	**−51.35** **(−8.7%)**
		Trabecular Apparent Bone Mineral Density (mgHA/cm^3^)	BMD_trab_	234.04	8.13	262.57	7.89	**0.004**	**−28.53** **(−10.9%)**
	Cortical Microstructure	Cortical Thickness (mm) - Direct	CtTh	0.35	0.02	0.39	0.02	0.107	−0.04 (−9.2%)
		Cortical Thickness _ SD (mm)	CtThSd	0.22	0.02	0.24	0.02	0.285	−0.03 (−11.1%)
		Cortical Bone Volume Fraction (%)	BV/TV_cort_	78.70%	1.11%	81.79%	1.11%	**0.020**	**−3.09** **(−3.8%)**
		Cortical Porosity (%)	CtPo	0.92%	0.07%	1.04%	0.07%	0.284	0.12 (−11.2%)
	Trabecular Microstructure	Trabecular Bone Volume Fraction (%) - Direct	BV/TV_trab_	35.03%	0.90%	38.14%	0.74%	**0.002**	**−3.10** **(−8.1%)**
		Trabecular Connective Density (mm^4^)	TbCD	6.45	0.32	7.05	0.37	0.210	−0.61 (−8.6%)
		Trabecular Structural Model Index (0–3)	SMI	0.95	0.10	0.58	0.10	**0.005**	**0.37 (63.5%)**
		Trabecular Number (1/mm)	TbN	2.18	0.07	2.34	0.05	*0.051*	*0.16* *(−6.9%)*
		Trabecular Thickness (mm) - Direct	TbNSd	0.206	0.003	0.208	0.003	0.651	- 0.002 (−0.8%)
		Trabecular Thickness _SD (mm)	TbTh	0.07	0.00	0.07	0.00	0.182	0.002 (3.4%)
		Trabecular Separation (mm) - Direct	TbSp	0.44	0.02	0.39	0.01	**0.011**	**0.05 (12.6%)**
		Trabecular Separation_SD (mm) - Direct	TbSpSd	0.24	0.02	0.18	0.01	**0.012**	**0.06 (32.7%)**
MH2 (n = 44, 22 pairs)	Volumetric Bone Density	Cortical Material Bone Mineral Density (mgHA/cm^3^)	TMD_cort_	800.40	9.53	832.97	8.52	**0.003**	**−32.57** **(−3.9%)**
		Cortical Apparent Bone Mineral Density (mgHA/cm^3^)	BMD_cort_	547.07	14.90	607.46	16.58	**0.002**	**−60.39** **(−9.9%)**
		Trabecular Apparent Bone Mineral Density (mgHA/cm^3^)	BMD_trab_	227.13	8.17	268.49	9.01	**0.000**	**−41.37** **(−15.4%)**
	Cortical Microstructure	Cortical Thickness (mm) - Direct	CtTh	0.35	0.01	0.38	0.01	*0.090*	*−0.03* *(−7.4%)*
		Cortical Thickness _ SD (mm)	CtThSd	0.20	0.01	0.19	0.01	0.937	0.001 (0.6%)
		Cortical Bone Volume Fraction (%)	BV/TV_cort_	80.03%	1.21%	83.77%	1.26%	**0.016**	**−3.74** **(−4.5%)**
		Cortical Porosity (%)	CtPo	0.92%	0.06%	0.96%	0.06%	0.626	−0.04 (−4.3%)
	Trabecular Microstructure	Trabecular Bone Volume Fraction (%)	BV/TV_trab_	33.69%	0.91%	38.17%	0.87%	**0.0002**	**--4.48 (11.7%)**
		Trabecular Connective Density (mm^4^)	TbCD	6.24	0.28	6.94	0.33	0.117	−0.70 (−10.1%)
		Trabecular Structural Model Index (0–3)	SMI	1.06	0.10	0.58	0.10	**0.0001**	**0.48 (84.1%)**
		Trabecular Number (1/mm)	TbN	2.08	0.07	2.28	0.06	**0.014**	**−0.20** **(−9.0%)**
		Trabecular Thickness (mm) - Direct	TbNSd	0.203	0.003	0.210	0.004	*0.090*	*−0.01* *(−3.5%)*
		Trabecular Thickness _SD (mm)	TbTh	0.07	0.00	0.07	0.00	0.445	−0.003 (−3.9%)
		Trabecular Separation (mm)	TbSp	0.47	0.02	0.40	0.01	**0.006**	**0.06 (16.0%)**
		Trabecular Separation_SD (mm)	TbSpSd	0.27	0.02	0.20	0.02	**0.008**	**0.07 (35.2%)**

BOLD: *P* < 0.05; *Italics*: *p* ≤ 0.10

NOTE: No adjustment for multiple independent statistical analyses of HR-pQCT outcomes

### HR-pQCT imaging - time effects

See Table 4 (Additional file 1) for details of the data values and results of the statistical analyses for the longitudinal time main effect analysis, representing the aging effect over 12-months for all participants independent of disease status. In summary, *density changes over time included*: Cortical bone material density at the MH3 site and UUD trabecular bone apparent density site were significantly reduced over 12-months (MH3 0.6% lower, UUD 2.5% lower). Changes in *cortical bone micro-structure over12-months included*: UUD cortical thickness and porosity significantly increased over 12-months (Thickness 4.7% greater; Porosity: 29.6% greater). *Trabecular bone micro-structure changes over 12-months included:* At the UUD site, trabecular number, connectivity and SMI all increased significantly across one year (Number: 1.9% greater, Connectivity 2.7% greater and SMI 3.4% greater). Whereas, trabecular thickness, trabecular spacing and variability were significantly lower (Thickness: 1% lower, Spacing 1.7% lower and variability 1.8% lower). Additionally, at the MH3 site, trabecular bone volume, thickness and thickness variation all increased significantly (volume 0.9% higher, thickness 0.7% higher and thickness variation 1.8% higher).

### HR-pQCT imaging - interaction (disease x time) effects

See Table 5 (Additional file 1) for full details of the data values and results of statistical analyses for the RA vs NRA by Baseline vs 12-months (interaction) main effect analyses. There was no significant interaction main effects for any cortical or trabecular bone density or microstructure outcomes examined, which indicates that the rate of change over 12-months was not different for the RA and NRA participants. Or alternately, that the RA participants did not show either an increased or decreased rate of change in bone micro-structure over the 12-months relative to the NRA participants, indicating as well that any underlying difference in bone micro-structure at baseline in the RA participants relative to the NRA participants persisted over the 12-months.

## Discussion

Despite marked improvements in early management of inflammatory joint symptoms, individuals living with RA continue to live with poor bone health and increased fracture risk compared to peers [2–6]. Our study is the first to explore prospective changes over 1-year in bone density and microstructure at the MH and DR in individuals recently diagnosed with RA who have been treated by a rheumatologist with care consistent with current practice guidelines. As we hypothesized, despite the introduction of DMARD (+/- oral glucocorticoids) medications at time of diagnosis, individuals living with early RA in this study demonstrated marked differences in periarticular trabecular and cortical bone density and microstructure, compared with NRA counterparts. Moreover, the pattern of very early micro-structural bone changes seen in those with newly diagnoses RA were consistent with changes more commonly seen in aging bone. After one year, the degree of detectable changes in periarticular density and microstructure that would normally occur with 1-year of aging, did not differ between individuals living with and without early RA. This speaks to early control of the disease activity with first line DMARD medication therapy (+/− glucocorticoids) that seemingly also mitigated any increased rate of systemic inflammatory mediated bone turnover, as the bone damage in RA did not worsen compared to NRA participants over 12-months. However, and again as we hypothesized, there was also no evidence of improvement in periarticular density or microstructure in the RA group. This suggests that the microstructural bone damage identified within the first year of diagnosis in the RA participants was resistant or very slow to recover despite achieving and maintaining relatively low levels of active joint inflammation and minimal self-reported functional limitation with the use of non-biologic DMARD therapy (+/- Glucocorticoid use).

Differences in periarticular bone density and microstructure seen in the first year following an RA diagnosis are notably consistent with changes in bone more commonly seen with aging in post-menopausal women and older adults of both sexes. As we age the homeostatic balance in bone remodeling in adulthood shifts to a negative imbalance, where bone is resorbed at a greater rate than it is replaced [38]. With aging, the pattern of bone loss in the cortical bone and trabecular bone regions is predictable. These changes include increased bone resorption at the endocortical bone surface leading to thinner and more variable thickness in the cortical shell. Cortical bone also becomes more porous and less materially dense [39, 40]. Trabeculae become thinner, more variable in thickness and become less connected resulting in more ‘rod-like’ compared with ‘plate-like’ structure as observed in younger bone [41]. These changes in the trabecular bone matrix also results in larger and greater variability in size of spaces between trabeculae [42]. Together, these age-related changes in cortical and trabecular bone microstructure ultimately results in a structurally weaker bone [43]. Moreover, that the age-related changes in cortical and trabecular bone microstructure reported in the literature are markedly similar to the pattern of cortical and trabecular bone changes we identified within the first year following an RA diagnosis. However, and importantly, that these apparent bone-aging changes were evident in both sexes and across all ages of RA participants. Suggesting that patterns of bone changes in early RA may be similar to the systemic hormonal mediated catabolic (negative) remodeling in bone commonly seen with aging, with the negative imbalance in bone remodeling in RA likely mediated more by proinflammatory cytokines that are known to be osteoclastogenic in nature, such as RANKL and Osteoprotegerin (OPG) [8, 44, 45].

Marked changes in bone microstructure a few months after a RA diagnosis implies that changes may have occurred rapidly at or around the time of inflammatory joint symptom onset and prior to response to RA medications. These changes may also have occurred over a longer period of time prior to onset of inflammatory joint symptoms. RA participants in our study were not imaged at the time of, or prior to the diagnosis. Thus, we are not able to discern which of these time-related factors have contributed to the early underlying changes in bone microstructure. However, as previously reported by Kleyer et al. (2013), individuals who are ACPA positive with no systemic inflammatory joint symptoms can show evidence of reduced cortical bone density and thinner and more porous cortices in the MH [27].

Our findings are largely consistent with previous cross-sectional studies exploring microstructural bone changes in individuals living with RA of longer duration compared to age and sex matched controls [18, 21–26]. Fouque-Aubert et al. (2010) [21], first reported lower trabecular bone density and trabecular thickness in the MH region in participants with an RA disease duration average of 9 years, as well as, lower trabecular thickness in the MH if a subgroup of early RA participants [Mean (SD) years: 1.0 (0.5)]. Subsequent cross-sectional studies have also examined HR-pQCT differences in periarticular bone in individuals living with RA for 8 or more years compared to controls and all have reported notable differences in bone density and microstructure in the MH or DR [18, 21–26]. Our study provides additional evidence of reduced bone density and altered cortical and trabecular bone microstructure in the periarticular MH and DR (UUD) regions in individuals with recently diagnosed RA that are consistent with age-related bone changes. Consistent periarticular micro-structural bone damage across all three regions of interest examined in our study also provides further indication that the peri-articular bone changes associated with early RA are likely systemically mediated to some degree. Furthermore, our findings indicate that worsened periarticular bone microstructure detected within a few months of inflammatory joint symptom onset persisted over 1-year despite early and clinically effective management of acute inflammatory joint symptoms with DMARD therapy, which may be a contributing factor to the persistent fracture risk in individuals living with RA despite more effective care with DMARD therapies [2–6].

Our study has several limitations. The first is the small size of the cohort, which may not represent the broader spectrum of individuals with RA in terms of severity of early symptoms or responsiveness to first line DMARD medications. In addition, the small cohort may have affected our ability to identify differences that may have existed due to lack of power related to large variability in some of the HR-pQCT outcomes evaluated. RA participants also received care from a small number of rheumatologist practicing in one large urban metropolitan region within Canada. Clinical care practices of these rheumatologists may not reflect practice patterns of other rheumatologists who provide care in other geographic regions or other health care systems or for individuals with limited access to timely specialist care [46–49]. We also only monitored change in bone microstructure over 1-year, which may have limited our ability to detect slower age-related changes. HR-pQCT has excellent precision (CV% varying from <1 to 3%) and is able to detect annual changes in many aspects of bone microstructure [19, 39, 42]. However, we may have needed a longer timeframe to identify measurable change in some aspects of bone quality, particularly changes in cortical bone density and microstructure given the greater imprecision with the evaluation of some aspects of cortical bone density and microstructure [19, 49]. We also did not use image analyses techniques to evaluate joint space or presence of periarticular erosions [18]. Nor did we utilize alternate methods for cortical bone segmentation and measurement of cortical porosity [50]. These HR-pQCT image evaluation approaches may have identified differences in periarticular bone and joint health in our participants that we were unable to detect with our methods. Finally, we did not examine the association between changes in bone quality and RA clinical factors, as this study was not designed or powered to secondarily explore the strength or direction of a potential relationship between RA disease factors (i.e. disease activity or ACPA status) or RA medications (i.e. DMARD only vs DMARD in combination with GC) and changes in microstructural bone quality. This would be an important avenue for future research considerations.

## Conclusions

Disadvantageous changes in trabecular and cortical bone density and microstructure occurs early in RA and are consistent with accelerated changes more commonly seen with aging bone. Moreover, these early changes in bone microstructure appear resistant or slow to recover despite clinically well controlled inflammatory joint symptoms with first line non-biologic DMARD therapies, with or without additional glucocorticoid medications. Although preliminary, findings suggest that further conservative or pharmacological treatments that address underlying bone health in early RA may serve to address the higher risk for fracture for those with RA compared with peers. Further research is also warranted to better understand ‘how early’ changes in bone microstructure may be associated with biomarkers of bone turnover in early RA and the natural progression of RA disease [51]. Research that evaluates factors associated with longer term loss in bone microstructure and fracture incidence in RA are also needed. Clinically, our findings support the importance of early and aggressive treatment and proactive monitoring and targeted management of bone health and fracture risk in individuals living with RA [52].

## Additional file

Additional file 1: Table S4. HR-pQCT Time (Baseline vs 12-months) Main Effects Summary. Summary of data and statistical results for the Time main (Baseline vs 12-months) effect analyses. **Table S5.** HR-pQCT Interaction [Disease (RA vs NRA) x Time (Baseline vs 12-months)] Main Effects Summary. Summary of data and statistical results for the Interaction (Disease x Time) main effect analyses. (XLSX 22 kb)

## Abbreviations

ACPA: Anti-Citrullinated Peptide Autoantibodies
ACR/EULAR: American College of Rheumatology / European League Against Rheumatism
Anti-TNF: Anti-Tumor Necrosis Factor
BMI: Body Mass Index
DMARD: Disease Modifying Antirheumatic Drug
FRAX®: Fracture Risk Assessment Tool (Registered)
HR-pQCT: High Resolution – peripheral Quantitative Computed Tomography
MH: Metacarpal Head
mHAQ: Stanford Health Assessment Questionnaire - Modified
NRA: Non-Rheumatoid Arthritis
RA: Rheumatoid Arthritis
RF: Rheumatoid Factor
SMI: Structural Model Index
UUD: Ultra-Ultra-Distal
